# The Potential of Plant-Produced Virus-like Particle Vaccines for African Horse Sickness and Other Equine Orbiviruses

**DOI:** 10.3390/pathogens13060458

**Published:** 2024-05-28

**Authors:** Kieran G. Pitchers, Oliver D. Boakye, Ivan Campeotto, Janet M. Daly

**Affiliations:** 1 One Virology, School of Veterinary Medicine and Science, Sutton Bonington, University of Nottingham, Nottinghamshire LE12 5RD, UK; kieran.pitchers3@nottingham.ac.uk; 2 School of Biosciences, Sutton Bonington, University of Nottingham, Nottinghamshire LE12 5RD, UK; oliver.boakye@nottingham.ac.uk (O.D.B.); ivan.campeotto@nottingham.ac.uk (I.C.)

**Keywords:** African horse sickness, virus-like particle, recombinant plant expression, *Orbivirus*, live attenuated vaccine

## Abstract

African horse sickness is a devastating viral disease of equids. It is transmitted by biting midges of the genus *Culicoides* with mortalities reaching over 90% in naïve horses. It is endemic to sub-Saharan Africa and is seasonally endemic in many parts of southern Africa. However, outbreaks in Europe and Asia have occurred that caused significant economic issues. There are attenuated vaccines available for control of the virus but concerns regarding the safety and efficacy means that alternatives are sought. One promising alternative is the use of virus-like particles in vaccine preparations, which have the potential to be safer and more efficacious as vaccines against African horse sickness. These particles are best made in a complex, eukaryotic system, but due to technical challenges, this may cause significant economic strain on the developing countries most affected by the disease. Therefore, this review also summarises the success so far, and potential, of recombinant protein expression in plants to reduce the economic strain of production.

## 1. Introduction

African horse sickness (AHS) is a potentially lethal disease of horses and mules, with other members of the Equidae family such as donkeys or zebras rarely exhibiting clinical signs. The disease is caused by African horse sickness virus (AHSV), a member of the genus *Orbivirus* of the family *Sedoreoviridae* [1]. The virus is transmitted by the *Culicoides* midge, mainly *C. imicola* and *C. bolitinos* species, and has devastated large portions of horse populations, with the most acute (pulmonary) form resulting in fatalities of over 95% of infected horses in some outbreaks. Due to the potential to cause high mortality and significant economic impacts, AHS is included in list A of notifiable diseases by the World Organisation for Animal Health (WOAH).

The origin of AHS is thought to be in Africa; it was described in 1569 by a travelling priest called Father Monclaro [2]. This disease remains a serious threat to equine populations with several sub-Saharan African countries (including Namibia, Botswana and Zimbabwe) having reported outbreaks in recent decades [3,4]. The largest known outbreak of AHS in South Africa was in 1854–1855 when approximately 70,000 animals were lost to the disease [5]. However, with the elimination of free-roaming zebras and the establishment of control and surveillance zones in South Africa, outbreaks are now much less common in the southern areas of the country [6]. Despite these factors, there are other areas that are seasonally endemic and multiple outbreaks do still occur in South Africa [7].

Although AHS is endemic only in parts of sub-Saharan Africa, it has caused outbreaks as far as Thailand to the east and Spain to the north. In these naïve populations, case fatality can reach over 95% [8]. During 1959–1961, AHSV outbreaks in countries such as Iran, Pakistan, Lebanon, and Turkey resulted in an estimated 300,000 equine deaths [9]. Outbreaks in non-endemic countries are a tangible threat with WOAH-designated disease-free countries such as Brazil, Paraguay, and Bolivia in South America determined by means of modelling to be suitable for the spread of the virus [10]. Furthermore, the closely related bluetongue virus (BTV), which has a similar vector, has been detected and overwintered successfully in more northerly territories in Europe, expanding its range of infection [11]. Finally, although zebras are sparse in non-endemic regions, horses, mules, and donkeys can develop sufficient viraemia to infect the vectors. This suggests that where there is a suitable vector present, if AHSV is introduced into a previously non-endemic region, an extended outbreak could occur with devastating consequences for the native populations of susceptible animals.

With over 300,000 horses in South Africa and their uses ranging from draught horses in rural communities to highly valued racing thoroughbreds, the burden of AHS is far-reaching [12]. For example, in South Africa, the horse-racing industry employs over 177,000 people, but exporting high-value animals to compete in international events, for example in Europe, is highly restricted. To export a horse from South Africa to an EU country, a 6-week quarantine is enforced and, increasingly, third-party countries such as Mauritius have been used for this purpose, but it is expensive [13]. Furthermore, if an outbreak occurs in the AHS-free zone in South Africa, then exports are totally suspended for a period of at least 2 years after the last reported case. Between 1997 and 2018, trade in live equids between South Africa and the EU was permitted less than half of the time [14]. For outbreaks in non-endemic countries, such as the most recent example in Thailand, there is a minimum of 2 years from the last outbreak until AHS-free status is regained [15]. This can have a serious impact on horse racing/breeding industries, and wild pony populations can also be adversely affected due to movement restrictions as part of the virus control strategies.

There is no specific treatment for AHS, but interventions such as non-steroidal anti-inflammatory drugs, antibiotics for secondary infections, and corticosteroids have all been used to provide supportive therapy. Prevention in endemic areas is mainly by vector control and vaccination with attenuated viral vaccines. While animal husbandry practices such as stabling horses before dark and in vector-proof housing seem to yield positive results, vaccination remains the most successful method of prevention (and control). The main vaccine used for protecting the horses in the sub-Saharan region against AHS is often referred to as a “live” attenuated vaccine (LAV). However, in AHSV-free regions, even where outbreaks have previously occurred, there is a reluctance to license attenuated virus vaccines due to concerns over genetic reassortment and reversion to virulence. Therefore, investigating novel vaccine strategies for AHS is an intensive area of research.

## 2. African Horse Sickness Virus

**Figure 1:**
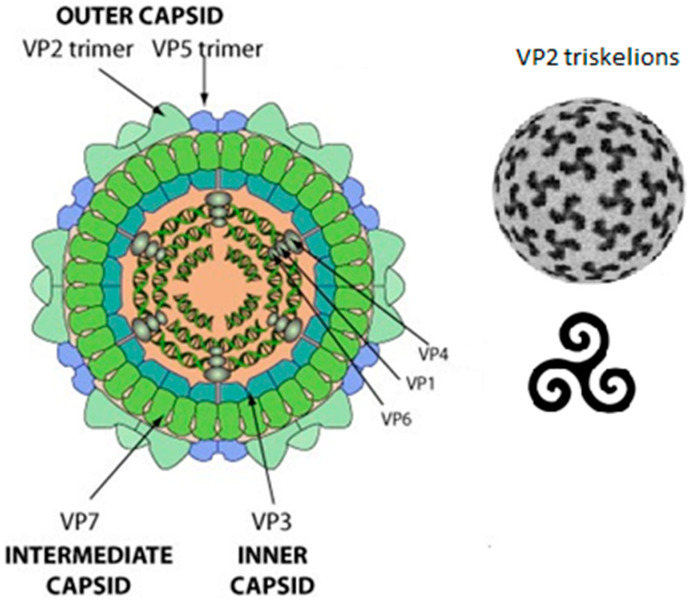
Schematic representation of AHSV viral particle. The outer capsid of the viral particle is made up of 180 monomers of triskelion-like trimers of VP2 [17] and 120 globular trimers of VP5. The core follows in two layers, 260 trimers of VP7 as the outer core and 60 units of VP3 homodimers as the inner core. The dsRNA genome is situated within the core together with the transcription complexes VP1, VP4, and VP6 [5]. Image adapted from VIPERdb (https://viperdb.org accessed on 3 March 2024) [17,22].

The AHS virion is a highly organised isometric non-enveloped particle, reported to be ±80 nm in diameter. The virion is symmetrical, quasi-icosahedral and almost morphologically identical to the *orbivirus* prototype, BTV [16]. It is made up of three concentric layers with the genome as the innermost layer, consisting of 10 segments of linear double-stranded RNA (Figure 1; [17]). These segments encode seven structural (four major and three minor) and five non-structural proteins. The four major structural proteins are arranged to create the inner core (VP3), outer core (VP7), and the capsid (VP2 and VP5) [18]. The inner core layer is made from 60 asymmetric dimers of VP3, the most conserved protein among the nine different serotypes of AHSV [19]. The three minor structural proteins remaining (VP1, VP4, and VP6) form a complex beneath the inner core, which is involved in transcription [16]. The inner core layer of VP3 is covered by 260 trimers of VP7, which are in turn covered by the outer layer of VP2 and VP5 trimers. The VP2 in the outer capsid is the target of most neutralising antibodies and determines the serotype of the virus, of which there are nine in total [17,20]. The outer layer of VP2/VP5 mediates the cell attachment and entry phase, therefore determining the host cell tropism. VP2 is thought to bind sialic acid moieties on cellular receptors, which then leads to ingress of the viral particle [21]. VP5 shares structural similarities with membrane fusion proteins, suggesting it helps mediate membrane penetration [21]. The current hypothesised model of infection suggests that viral entry is endosomally mediated, with the low pH causing VP2 to dissociate from VP5, which allows VP5-facilitated membrane permeabilization and entry of the viral particle into the cytoplasm.

As well as the seven structural proteins, there are a further five non-structural proteins synthesized in infected cells and involved in viral replication, assembly, and egress: NS1, NS2, NS3, NS3a (which lacks the 13 N-terminal amino acids of NS3), and NS4. The first non-structural protein, NS1, forms a tubular structure within the cytoplasm and is also involved in the up-regulation of viral protein synthesis [23]. The viral inclusion bodies observed are composed of NS2, which binds single-stranded RNA, thereby promoting viral replication and subsequent core assembly [24]. Once assembled, viral particle release is facilitated by NS3 and NS3a (although it is not essential); these two proteins are considered to be the only membrane proteins that are glycosylated [25,26]. The last non-structural protein, NS4, is thought to counteract the interferon response of the host to minimize the effectiveness of the innate immune response [27].

## 3. African Horse Sickness Disease

**Figure 2:**
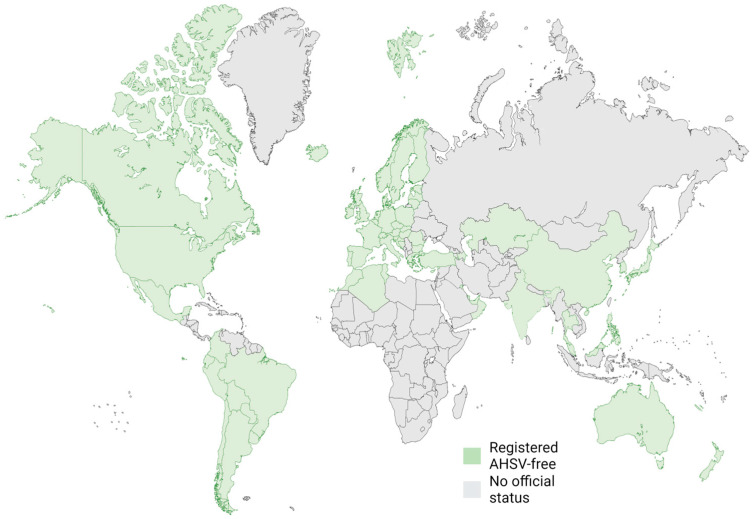
Map showing African horse sickness virus status of countries worldwide. Adapted from https://www.woah.org/en/disease/african-horse-sickness/ accessed on 3 March 2024. Created with BioRender.com.

The pulmonary form of AHS, or “dunkop”, as it is colloquially known in South Africa, causes clinical signs such as fever, congestion, and difficulty breathing. Often, there are large amounts of fluid discharged from the nostrils following pulmonary oedema, with most cases culminating in death. With case fatality approaching 100%, this has a significant impact on susceptible equine populations, usually naïve animals such as those unvaccinated in disease-free areas (Figure 2; [8]). The cardiac presentation, “dikkop”, causes a fever and congestion of the mucous membranes leading to a swollen head due to subcutaneous oedema. The cardiac form has a case fatality of approximately 50%, while the mixed form, a combination of signs from both the pulmonary and cardiac, has an average mortality between the two presentations (around 70%). The final form of the disease (febrile) is usually only associated with a fever, followed by full recovery. In susceptible horses, the mixed presentation is the most common [28]. However, partially immune horses are likely to develop the febrile form of the disease while zebras, which are the natural reservoir of the virus, are sub-clinically affected.

The primary pathogenesis of the disease is consistent across the different presentations. After being bitten by an infected vector, replication initially takes place in a nearby lymph node before the virus is disseminated around the body via the circulatory system by associating strongly with erythrocytes [29]. The virus has been primarily isolated from the heart, lungs, spleen, and lymphoid tissues, where it penetrates the endothelial cells, but monocyte–macrophage cells can be involved [30]. For susceptible animals, such as horses, the period of viraemia is usually between 4 and 8 days but with donkeys it can be extended up to 28 days [31]. In zebras, viraemia can last for up to 40 days post-infection [32].

Presumptive diagnoses of AHS can be via clinical signs and post-mortem lesions. To achieve a definitive diagnosis, laboratory confirmation of the virus is required, with serotyping also advised for surveillance purposes and vaccine monitoring. This is accomplished through reverse transcription–PCR assays with blood sampled from live animals, ideally at the peak of infection, or spleen and lung samples from recently dead animals [33].

The transmission of AHS is by biting midges, principally of the genus *Culicoides*. The primary vector is thought to be *C. imicola*, which is common throughout Africa, Southeast Asia and southern Europe [34]. There have been studies reporting the ability of other members of *Culicoides* to transmit AHS, such as the BTV vector *C. variipennis* (*sonorensis*) [35]. Moreover, during an outbreak in Spain, AHS was isolated from other known vectors of BTV, *C. obsoletus* and *C. pulicaris* [36]. This is concerning due to the recent intrusion of BTV into Europe. Like BTV, it is possible for AHSV to overwinter in southern Europe. This is evidenced by the AHSV outbreak in Spain, which lasted 3 years, until AHSV was eradicated in 1990 with the help of vaccination, but not before the culling of approximately 3,000 equids [37]. This outbreak was thought to have been caused by the importation of infected zebras. Tighter restrictions on live animal imports from Africa have prevented any further outbreaks. Another species of biting midge, *C. bolitinos,* whose larvae are found exclusively in cattle dung, has been implicated as a vector [38]. This is a potentially complementary habitat condition to the primary vector and may explain outbreaks of AHS in more mountainous, quick-drying terrain such as in Lesotho. Other arthropods have been demonstrated to transmit AHSV in laboratory settings, with both mosquitoes and the camel tick *Hyalomma dromedarii* theoretically able to transmit the virus, but the importance of these as vectors in an outbreak of AHSV is thought to be negligible [39].

## 4. Current Vaccination Tools for AHS Prevention

The attenuated virus vaccine used throughout the endemic regions as a primary means of disease control was developed from work in the 1930s at the Onderstepoort Veterinary Research Institute, South Africa. The vaccine is supplied by Onderstepoort Biological Products as separate trivalent (serotypes 1, 3, 4) and tetravalent (serotypes 2, 6, 7, 8) doses given 2–3 weeks apart. Serotype 5 was originally included in the formulation but due to concerns over residual virulence, it was removed. Serotype 9 was not included as it was not prevalent in southern Africa and was considered unnecessary. Despite this, there does seem to be heterologous protection rendered by the other serotypes against those two missing serotypes. Cross-protection has been documented in laboratory settings between serotypes 1 and 2, 3 and 7, 5 and 8, and 6 and 9 [5,40]. However, it is unknown to what extent the heterologous protection extends to natural infections.

Due to the segmented nature of the AHSV genome, there is always a risk of genetic reassortment between vaccine and circulating viruses leading to a reversion to virulence, which has been found to be the cause of at least three outbreaks of AHS [41,42]. There is also a concern that the vector may transmit AHSV vaccine strains, potentially leading to circulating vaccine strains increasing the chances for genetic reassortment [43]. To reduce the risk of this, vaccination in South Africa may only occur between 1 June and 31 October as the vector is less active during this period.

In regions where AHSV is not endemic, monovalent inactivated virus vaccine preparations are preferred for control of outbreaks of the virus after it has been typed. However, as both attenuated and inactivated virus vaccines contain the whole virus, vaccinated animals cannot be differentiated from those that have been infected using serological tests. Being able to use tests that differentiate infected from vaccinated animals (DIVA) would undoubtedly help with the import/export of high-value horses, in which there is a significant trade. The requirement for high levels of containment to propagate AHSV prior to inactivation also adds to the cost of the production of inactivated virus vaccines.

## 5. Use of Virus-like Particles as a Vaccination Strategy

Currently, to achieve disease-free status from the WOAH for AHS, a country cannot allow systematic vaccination (Figure 2). There are also no vaccines licensed by the European Medicines Agency for use in the EU due to safety concerns with the attenuated vaccines used elsewhere. Therefore, there is a need to replace the pan-serotype vaccine currently used in endemic areas and monovalent preparations used for outbreaks. One promising avenue of research is the use of virus-like particles (VLPs). These consist of a core molecule that can be used to display an antigen of choice, which mimics a viral particle, but with no genome. As VLPs do not contain a genome, they do not replicate and are, therefore, inherently safer. They can elicit strong immune responses as they stimulate both cellular and humoral immune responses [44,45]. Displaying multiple antigens in proximity offers the potential to create a significant immune response, which is difficult to achieve with monomeric viral proteins even with the use of potent adjuvants.

The first recombinant human VLP-based vaccine was developed in 1986 against the hepatitis B virus, after the successful expression of the viral surface antigen in yeast cells (HBsAg; Recombivax HB^®^, Merck & Co., Inc., Rahway, NJ, USA). Other examples of commercial VLP vaccines include Hecolin^®^ (Wantai BioPharm, Beijing, China), which protects against hepatitis E virus and involves bacterial expression, and Cervarix^®^ (GlaxoSmithKline, Middlesex, UK), which protects against human papillomavirus and is also expressed in yeast cells. Cervarix^®^ uses the self-assembling L1 structural protein and has been shown to be effective in controlling this common sexually transmitted virus, a known risk factor for cervical cancer in humans [46]. Currently, VLPs derived from over 20 different human viruses, including HIV, SARS-CoV-2, and Ebola, are at various stages of development [47].

In veterinary medicine, VLP vaccines have been developed against viruses that infect various animals including rabbits, chickens, sheep, horses, and fish [47]. In chickens, VLPs derived from influenza A virus or infectious bursal disease virus both outperformed the commercial inactivated vaccines by providing better protection [45,48,49].

In the event of an outbreak of AHSV, a vaccine preparation that can be easily altered based on the serotype would be commercially attractive. In this area, VLP technologies can make great improvements compared to attenuated vaccines, which can take many passages in different cell lines to reach effective attenuation by traditional methods or require the introduction of attenuating mutations using a plasmid-based reverse genetics system such as that developed by Matsuo et al. [50], which is technically demanding. Systems such as SpyCatcher/Tag, which is based on a protein from *Streptococcus pyogenes*, offer potential as a “plug-and-play” technology [51,52,53]. The protein tag (SpyTag) is fused to the target antigen, while the interacting domain (SpyCatcher) is exposed on the acceptor VLP monomer. When the two protein sequences are in proximity, they form an irreversible and stable covalent bond. The VLPs display 60 sites, which can theoretically be all occupied by the antigen, although steric hindrance and antigen properties limit total occupancy. To change the displayed antigen, a new protein is expressed recombinantly with the appropriate SpyTag and linked to the desired VLP. There are several VLP variants, which have been improved by mutagenesis or computationally, such as SpyCatcher003-mi3 [54,55,56], and they have been used successfully to produce vaccine prototypes against malaria, influenza, and more recently SARS-CoV-2 [57,58].

Recombinant viral proteins can be expressed in over 170 different expression systems including yeast, bacterial, insect, mammalian, and plant cells [59,60,61]. The type of expression organism used is important because viral proteins are complex molecules that require certain factors to be efficacious. Such factors include appropriate post-translational modifications and correct folding of the protein [62,63]. Other factors such as levels of protein expression, scalability, production, and maintenance costs contribute to how economically feasible an expression system is [64]. Each expression system has individual characteristics; bacterial cells are usually easy to scale up, yet they present safety concerns due to a possibility of endotoxin contamination and acetate accumulation. Bacteria also lack the post-translational machinery necessary for authentic eukaryotic protein modifications, especially glycosylation, which is often key for antigenicity [65]. A disadvantage of yeast cell expression is the potential for the “hyperglycosylation” of proteins due to high levels of mannose modification, and their lack of mammalian-like post-translational modification limits their use for generating non-enveloped VLPs [66]. Insect cells require a longer procedure to express protein constitutively or transiently, and they typically have low levels of protein expression and produce glycosylation patterns different from that observed in mammalian cells [67,68]. While mammalian cells provide correct protein folding and post-translational modifications and often yield adequate levels of recombinant proteins, they are expensive to maintain and require stringent sterility conditions [69]. As a result, plant expression has been cited as a cheaper, more scalable alternative, which produces a high quality and quantity of functional recombinant proteins for the biopharmaceutical industry (“pharming”) [38,70,71]. Most plant pathogens pose a negligible threat to human or animal health, so this reduces the risk of harmful infectious agents within the vaccine. They also do not require animal-derived reagents in their culture, unlike some mammalian cell cultures.

## 6. Plant Expression

There are two main approaches to enable recombinant protein production in whole plant systems, depending on whether the protein expression is constitutive or not. There are plant cell suspension and cell-free lysate systems that have also been areas of research, but these are outside the scope of this review, although there is literature available on advancements in that area [72,73,74]. Transient expression systems cause a grown wild-type plant to start producing recombinant proteins, while transgenic plants are stably transfected and will continuously produce the protein without an induction system. The transient approach has become more widely used as it does not require the time-consuming process of creating a stably transformed plant. This time constraint of the transgenic approach means it is not as suitable for responding to emerging epidemics or rapid screening. Furthermore, continuous production of foreign proteins can be detrimental to the development of stably transformed plants.

**Figure 3:**
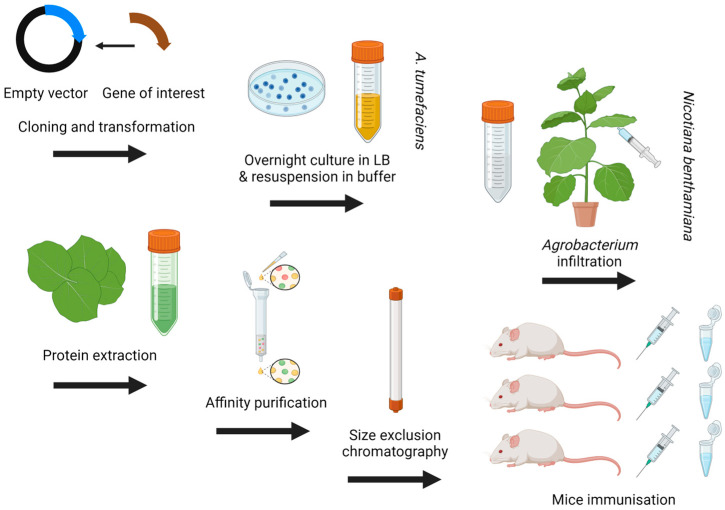
Plant protein expression methodology for small-scale trials. Created with Biorender.com.

The different transient approaches all use the soil bacterium *Agrobacterium tumefaciens*, and the expressed protein can be changed by simply cloning in a different gene of interest. The wild-type *A. tumefaciens* can insert a section of DNA (T-DNA), from its tumour-inducing (Ti) plasmid, into a susceptible plant, where it is then expressed in the host cell, causing crown gall disease. To attenuate the wild type, the Ti plasmid was cured. A recombinant plasmid with a new section of T-DNA containing the gene of interest can then be inserted. The replacement plasmid should contain certain elements, such as border sequences, to allow the new T-DNA to be transposed into the plant cell. Other elements include untranslated regions, which flank the borders and up-regulate the transposition of the T-DNA, a gene-silencing suppressor such as P19, which prevents down-regulation of the target gene during expression in the plant cell, and a bacterial origin of replication to allow propagation in the *A. tumefaciens* host. Once a gene of interest is inserted into the T-DNA region of an appropriate plant expression vector, the plasmid can be transformed into a suitably attenuated *A. tumefaciens* host. Arguably, currently, the most popular plant expression vectors for the transient expression of proteins in plants are the pEAQ vectors. These use modified sequences from cowpea mosaic virus (CPMV) to boost translational efficiency, referred to as CPMV-hypertrans or CPMV-HT [75]. A culture of the bacterium is then infiltrated into the plant leaves by hand via a syringe or at scale by submersion, facilitated by a negative air pressure. After 5–9 days, the leaves are harvested, and the recombinant protein is extracted and purified (Figure 3).

The first VLP expressed in plants was the surface antigen of hepatitis B virus (HBsAg) using transgenic tobacco, the same antigen produced in a yeast expression system for Recombivax HB^®^ [76]. Since these initial efforts, significant advancements have been made with recombinant protein expression in plants, and improvements in the transient expression system mean that VLP vaccines produced in plants now have the potential to compete with current, commercially produced vaccines. For example, promising initial results were obtained when the capsid protein of porcine circovirus type 2 (PCV-2), which self-assembles into VLPs, was expressed in plants using the pEAQ-HT vector. The VLP vaccine-induced immune responses in mice were comparable to if not greater than the commercially available recombinant protein vaccine Ingelvac CircoFLEX^®^ [77].

Commercial interest in the plant expression platform has increased significantly in the past decade. The Canadian-based company Medicago (now Aramis Biotechnologies, Quebec City, QC, Canada) produced the first VLP-based vaccine approved for human use; Covifenz^®^ was a COVID-19 vaccine formulated with an adjuvant manufactured by GlaxoSmithKline. Work to develop an influenza A virus (IAV) vaccine provided the foundation for the development of Covifenz^®^, which involves the expression of the spike protein. During the development of IAV vaccines expressing the haemagglutinin (HA) envelope glycoprotein, the rapidity with which the plant expression system could be used to respond to emerging novel subtypes was demonstrated. It took only 3 weeks from the release of the HA sequence of the 2009 H1N1 pandemic virus to obtain purified VLPs that were subsequently shown to be immunogenic in mice [78]. Similarly, from the first reported human case of an avian H7N9 IAV in China on 29 March 2013, for which the HA sequence was subsequently made public, it took only around 7 weeks to demonstrate the immunogenicity of a plant-expressed VLP in mice [79].

Other companies that are using plant-based expression systems include Leaf Expression Systems, who have the exclusive rights to sub-license the CPMV-HT system (pEAQ vectors). Icon Genetics developed the “magnICON^®^” system based on tobacco mosaic virus, which uses co-infiltration of *Agrobacterium,* separately expressing three modules. The 5′ module contains the promoter, viral polymerase, and movement protein, while the gene of interest is cloned into the 3′ module. The third, recombinase, module fuses the 5′ and 3′ modules in the plant cells so that they assemble into an RNA replicon vector that can move cell-to-cell [80]. A phase I trial of a norovirus VLP vaccine produced using this system was recently published [81]. More recently, the same group that developed the CPMV-HT system developed a novel expression vector called pHREAC (High Recombinant Expression Associated with CPMV) that has a rationally designed synthetic 5′ untranslated region (UTR) with the 3′ UTR of CPMV that is also intended to be made available for use by resource-poor entities [82].

Researchers have begun investigating replacing vaccines based on the traditional cell-based methods against diseases caused by the *Orbivirus* genus with VLP-based preparations. As for AHSV, there are two types of commercial vaccines against BTV currently available, inactivated and attenuated. The attenuated vaccines have similar disadvantages to the currently available attenuated virus vaccine for AHSV and so are not used in Europe; the inactivated vaccines do not have concerns about genetic reassortment but are largely serotype-specific [83]. In contrast to the enveloped viruses IAV and SARS-CoV-2 that only require expression of a single protein, co-expression of the four structural capsid proteins (VP2, VP3, VP5, VP7) in the plant system was used to produce a BTV VLP [84]. The VLP provided protective immunity to sheep challenged with BTV after immunisation. To produce a pan-serotype vaccine to compete with the current multivalent attenuated virus vaccine, chimeric VLPs have been produced by using heterogenous VP2 during assembly [85,86]. The production of chimeric VLPs reduces the number of constructs necessary to achieve a multivalent vaccine, by swapping only a single viral protein for each serotype. When inoculated into sheep, the plant-based chimeric VLPs showed long-lasting serotype-specific neutralising antibodies, equivalent to the monovalent attenuated virus vaccine [87,88].

In the last decade, the first AHSV VLP, for which codon-optimized genes for VP2, VP3, VP5, and VP7 of a serotype 5 virus were cloned into the pEAQ-HT plant expression vector, has also been produced in plants [87]. Not only was production determined to be a fast method, and effective, with the VLPs able to neutralise infectious virus in cell-based assays, but also economically viable to produce. The plant-produced VLPs were also reported to be safe to use in horses and produced comparable neutralisation tires to those obtained with the AHS attenuated virus vaccine [89].

**Figure 4:**
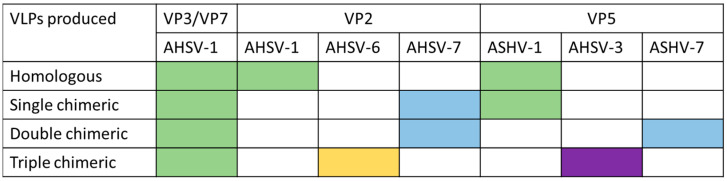
African horse sickness virus (AHSV) chimeric virus-like particles (VLPs) produced in Rutkowska et al. [89]. The box colour highlights which serotype the constituent proteins for each VLP construct are derived from: green = serotype 1, blue = serotype 7, yellow = serotype 6, and purple = serotype 3.

The generation of chimeric VLPs made from AHSV capsid proteins was also explored using the CPMV-*HT* system [86]. To more efficiently generate VLPs, the VP3 and VP7 of AHSV serotype 1 were expressed from the same plasmid. They co-expressed the VP2 and VP5 of AHSV-1 and various combinations of VP2 and VP5 from other AHSV serotypes to generate chimeric VLPs (Figure 4). Only the triple chimeric construct was used to immunise horses, and this induced low titres of neutralising antibody against the AHSV-6 virus from which the VP2 sequence was derived, although so did the commercially available attenuated virus vaccine. The same group also generated a double-chimeric VLP with AHSV-1 VP3/VP7 andVP2 and VP5 of AHSV-5, which protected all six IFNAR^−/−^ mice immunised with the VLP against a lethal AHSV-5 challenge [90].

Recently, it has been described that a nonavalent vaccine preparation of plant-produced VP2 has elicited high titres (≥112) of neutralising antibodies in IFNAR^−/−^ mice against all nine serotypes of AHSV after just two vaccinations [91]. A first trial using chimeric AHSV-1/5 and AHSV-1/6 VLPs as quadrivalent primary and booster vaccines with the VP2 antigens from serotype 1 and the other unrepresented serotypes failed to elicit comparable neutralising titres. However, the reduced titres could be due to an overall decrease in the amount of each VP2 antigen displayed to the immune system. Potentially, the nonavalent preparation could be replicated with all nine serotypes of VP2 displayed on VLPs and increase the immune response observed.

However, there are issues regarding plant expression: low yields, inconsistent product quality, and difficulties when scaling up. Despite this, one techno-economic model still suggested that the use of a hydroponic system for the growth of *Nicotiana benthamiana* and the recombinant production of monoclonal antibodies could reduce costs by over 50% when compared to traditional mammalian systems [92]. However, these cost savings might be product-specific, and more work needs to be done on the comparative economics when more data are available [93].

Protein instability due to protease activity and the difficulties in directing protein expression to a suitable compartment within the plant cell can also contribute to lower productivity. There has been some success with both genetic and physical ways of increasing this productivity, but more work is still needed to improve the consistency of yields [94,95,96]. Due to the use of whole plants as an expression system, there is substantial downstream processing needed to extract the expressed protein. This is a difficult aspect to upscale and make commercially viable, but initial research has been performed in this area with some success [97]. The final barrier to a widely used plant expression system is related to the subject of the protection of intellectual property; there is a lack of freely accessible vectors and host plants with supported protocols. This is especially true for small-scale commercial enterprises, but the use of resources such as OpenMTA and contributions to it by researchers could remove this obstacle [82,98].

## 7. Concluding Remarks

The traditional cell-based vaccine preparation strategies, while effective compared to the pre-vaccination era, may not present the best solution for future disease outbreaks. Attenuated virus vaccines pose safety concerns, and inactivated virus vaccines are often not very effective for reasons such as denatured antigenic regions. As horses are regarded as both a high-value commodity (e.g., for breeding and competitions) and a companion animal, there is a market for a new vaccine, with owners likely to pay for it to protect valuable animals. This could spur investment in the development of new vaccines, especially as plant production is considered relatively low-cost, with additional benefits when compared to mammalian or insect cell recombinant expression.

There have been other approaches to develop a safer, more efficacious AHSV vaccine; strategies such as using modified vaccinia Ankara virus (MVA) or reverse genetics (RG) have shown potential. An MVA vaccine preparation developed by Castillo-Olivares et al. has shown great promise, with the VP2 of serotype 4 (MVA-VP2) inducting neutralising titres in ponies with limited adverse reactions [99]. Further studies investigating MVA as a vehicle for vaccine preparations, based on a single VP2 serotype, showed its ability to protect both horses and IFNAR^−/−^ mice from a homologous AHSV challenge with no detectable viraemia [100,101]. There is also some evidence that the use of the more conserved NS1 protein in the vaccine preparation can provide a level of cross-protection across the serotypes, but this is unconvincing, and more research is needed with heterologous serotypes that are lethal in the IFNAR^−/−^ mouse model used, and dissection of what the MVA-NS1 preparation actually contributes to the level of immunity [102,103].

The data show that MVA has potential for a vaccine against AHSV, but there is yet to be a pan-serotype preparation that elicits significant cross-protection. Furthermore, repeat injections of MVA vectors can lead to diminished immune responses to later doses due to immunity to the vector. However, this has not been described yet for the AHSV MVA vaccines, and there are options for using different vectors if needed, such as an adenovirus. MVA vaccines also employ a costly cell culture, which is one of the main advantages of the plant expression systems described. The issue of transgene stability must be addressed with MVA vaccines, which may affect the development and scaling up of vaccine preparations based on these [104,105].

Reverse genetics (RG) vaccines are another promising avenue of research into a replacement AHS vaccine; these inoculations cause the host to produce virus particles missing a critical protein for replication or viraemia. These are often categorized into two platforms: entry-competent replicative–abortive (ECRA) or disabled infectious single animal (DISA). The ECRA vaccines cannot complete their replication cycles, while the DISA ones prevent viraemia and transfer to other cells. Recent work on an AHSV RG vaccine established a DISA-based preparation that contained a deletion in the NS3/NS3a gene. However, this showed high variability in its protection of horses from a highly virulent AHSV-4 strain [106]. On the other hand, a more recent ECRA formulation provided complete protection from mortality against the same strain in vaccinated horses [107]. It elicited neutralising antibodies against multiple serotypes when a multivalent formulation was used, and viraemia after a challenge was consistently low in all but one animal. However, it failed to raise neutralising antibodies against AHSV-5, which was not included in the multivalent formulation, so further research is needed to determine if a comprehensive multivalent AHSV RG vaccine is possible. Furthermore, while the risks of recombination with circulating field strains of AHSV are minimal, it is still a possibility. Therefore, comprehensive environmental risk assessments are needed, which are not required for plant-based VLP vaccines.

With a warmer climate, the incursion of AHSV into southern Europe and elsewhere may become more commonplace, with environmental conditions more suitable for insect vectors and viral transmission. Outbreaks of *orbiviral* species in non-endemic regions pose a serious threat, such as the recent BTV expansion in Europe, as there are often multiple serotypes or strains, which complicates preparation strategies. Peruvian horse sickness virus (PHSV), for example, is a more recently discovered *orbiviral* species. The Peruvian horse sickness virus is thought to be transmitted by mosquitoes and has been found in Peru, Brazil, and within the northern territory of Australia [108]. Furthermore, the equine encephalosis virus (EEV) was found to be circulating within Israel when it was thought to be only endemic to southern Africa. EEV is another *Orbivirus* spread via *Culicoides* midges, and while the disease caused is milder than AHS, the implications for the potential for AHSV to spread to similar regions are significant.

The emergence and re-emergence of orbivirus diseases across the world requires vaccine technologies that are adaptable, enabling a timely response to outbreaks. While the impact of PHS on equids is still relatively unknown, using sequence data, it could be possible to add domains of putative immunological regions onto existing VLP scaffolds to create a vaccine. The potential of VLP vaccines to facilitate this “plug-and-play” functionality offers a rapid-response capability, and the use of a plant expression system can ensure that production is not limited to expensive facilities, which limits access for developing countries where the need may be greatest. VLPs also provide further benefits such as being DIVA-compliant, possibly offering relaxed equid import/export requirements for both AHSV-endemic countries and those with periodic outbreaks.
